# Eye Movement Impairment Recovery in a Gaucher Patient Treated with Miglustat

**DOI:** 10.1155/2010/358534

**Published:** 2010-09-26

**Authors:** Agostino Accardo, Stefano Pensiero, Giovanni Ciana, Fulvio Parentin, Bruno Bembi

**Affiliations:** ^1^Department of Electronics (DEEI), University of Trieste, 34127 Trieste, Italy; ^2^Department of Ophthalmology, IRCCS “Burlo Garofolo”, 34137 Trieste, Italy; ^3^Metabolic Diseases Unit, IRCCS “Burlo Garofolo”, 34137 Trieste, Italy

## Abstract

In Gaucher Disease (GD) the enzyme (imiglucerase) replacement therapy (ERT) is not able to stop the progression of the neurological involvement, while the substrate reduction therapy (SRT), performed by N-Butyldeoxynojirimycin (miglustat), is an alternative that should be evaluated. Two sisters, presenting the same genotype (R353G/R353G), were diagnosed as suffering from GD; one of them later developed neurological alterations identified by quantitative saccadic eye movements analysis. The aim of the study was to quantitatively measure the miglustat effects in this GD neurological patient. Eye movement analysis during subsequent controls was performed by estimating the characteristic parameters of saccadic main sequence. The study demonstrates that the SRT alone can be effective in GD3. Moreover, it confirms that quantitative eye movement analysis is able to precociously identify also slight neurological alterations, permitting more accurate GD classification.

## 1. Introduction

Gaucher disease (GD) is an autosomal recessive lysosomal glycolipid storage disorder characterized by the accumulation of glucocerebroside (glucosylceramide) in reticuloendothelial cells [1].

The gene coding for the deficient enzyme glucocerebrosidase (acid beta-glucosidase) is located on chromosome 1q21 [2].Three phenotypes are traditionally recognized based on the absence (type 1) or presence and severity (types 2 and 3) of CNS involvement. Specific mutations in the beta-glucocerebrosidase gene are associated with specific clinical presentations, for example, the L444P mutation produces neurologic involvement.

The liver, the spleen, and long bones are the primary organs affected by the storage of glucosylceramide, mainly derived from the normal turnover of leukocytes and erythrocytes. The highly cytotoxic substance glucosylsphingosine (the nonacyl derivative of glucosylceramide) is also stored in excess in the viscera and in the brain, leading to cell death. Such neuronal destruction involves mainly the brain stem and deep cerebellar nuclei, but the thalamus, basal ganglia, and spinal cord are also affected.

Type 3 (GD3) represents the subacute, juvenile neuronopathic form, with onset in the teenage years and a chronic course. The severity of GD3 is intermediate between type 1 (GD1) and type 2 (GD2) with milder neurological features. The first symptoms are due to the massive visceral involvement, and disorders of eye movements are the usual presenting signs [3].

Adult patients [4, 5] in whom symptoms had begun in late childhood, adolescence, or early adult years present myoclonic epilepsy [6] and a distinctive supranuclear eye movement disorder affecting primarily horizontal gaze and only occasionally vertical gaze [7–9]. The early defect in horizontal gaze involves the saccadic system, and the disorder mimics closely congenital ocular motor apraxia [10]. Ocular motor abnormalities include horizontal saccadic initiation failure (SIF), with blinking, strabismus, slow horizontal and downward saccades, and an abnormal vestibulo-ocular reflex [10]. Horizontal SIF is the most consistent finding and is frequently the first sign of neurological involvement [11]. Vertical SIF usually indicates a progression of the disease, even if one case with vertical without horizontal SIF has been pointed out [11]. Both quick phases of optokinetic nystagmus [12] and voluntary saccades [13] can be used to early detect and follow the neurological involvement.

The phenotypic continuity between nonneuronopathic and severe acute neuronopathic forms of Gaucher disease (GD) is emerging from the literature [14–16], contrary to a clear-cut distinction among the classical GD1, GD2, and GD3 types. In a large series of French patients, the clinical characteristics suggest that the three forms of GD each involves a different profile of neurological manifestations [17].

GD1 is treatable with appropriate amounts of exogenous enzyme (imiglucerase) replacement therapy (ERT), whose safety and efficacy have been clearly documented [18]. Splenectomy is rarely necessary, since specific treatments have recently become available. ERT is however unable to stop the progression of the neurological involvement. Due to the fact that infusion of glucocerebrosidase increases enzyme activity in the CNS if a dosage of 120 U/kg body weight or higher is given [19], the role of such high-dose ERT in neuronopathic cases was studied: it was concluded that the latter is not able to stabilize neurological disease [20].

To protect the brain of GD patients, substrate reduction therapy (SRT) performed by N-Butyldeoxynojirimycin (miglustat) could be an alternative. The formation of glycosphingolipids is decreased to amounts that may be metabolised by the residual enzyme [21]. In patients with visceral GD, the efficacy of SRT with miglustat has been demonstrated [22], while the benefit of miglustat also for patients with neuronopathic GD is not proved, given the contrasting results obtained [23, 24].

In this paper, the clinical history of two GD sisters, initially treated with ERT, is described. One of them developed saccadic eye movement alterations that disappeared after two years of miglustat therapy.

## 2. Case Report

Two sisters (F. I. and A. I.), out of 7 siblings (5 females, 2 males), offspring to second cousin parents, presenting the same genotype (R353G/R353G), were GD diagnosed in 1983 and both submitted to splenectomy in the same year. During adolescence, they presented epilepsy responsive to barbiturate: generalized tonic-clonic seizures (patient F. I.) and partial complex seizures (patient A. I.). A first saccadic eye movements recording was carried out in both patients in year 2000, during their annual clinical control. The eye movement recording was repeated in 2005 and in 2007.

F. I., born in 1961 and suffering from hepatosplenomegaly, anemia, thrombocytopenia, and bilateral necrosis of the femoral epiphysis at the moment of diagnosis, began ERT in 1995 (alglucerase/imiglucerase: 30 U/kg every 2 weeks). At that time she showed liver cirrhosis without signs of liver insufficiency; focal spike activity at EEG (left temporal region); normal visual evoked potentials (VEP) and auditory brain responses (ABR); increased threshold intensity and reduced amplitude at upper limb motor-evoked potentials. The 2005 control brought out that in the previous 5 years ERT therapy had been performed without continuity; therefore, treatment was changed to SRT miglustat (200 mg, 3 t.i.d) oral therapy. The barbiturate therapy continued. At the 2007 control, liver volume remained stable, no bone fracture was reported or detected, and the EEG proved unchanged (infrequent focal paroxysmal discharges). The miglustat treatment was confirmed owing to the absence of adverse events (gastrointestinal disturbances) in the two years of oral treatment.

A. I., born in 1967 and suffering from splenomegaly, anemia and thrombocytopenia at the moment of diagnosis, began ERT in 2002 (regularly performed) because of a hepatic involvement (imiglucerase: 15 U/kg every 2 weeks). At that time she did not manifest neurological signs and had normal EEG and ABR, so that the barbiturate therapy was stopped. The 2005 control evidenced diffuse bone pain. She changed the treatment to SRT miglustat (100 mg, 3 t.i.d.) too, oral therapy, that was confirmed at the 2007 control, thanks to the tolerability of the oral treatment.

Saccadic movements of both eyes were recorded by means of the infrared *limbus tracking* technique (a very accurate technique in cooperative patients), with the subject looking at a target (a red light spot) in random horizontal motion, in a visual range of ±15 deg with amplitudes of 5, 10, 15, and 20 deg. Two 70-movements tests were performed, at a 30-minute interval. In both patients, the ophthalmic and orthoptic examinations, performed before each saccadic test, showed the absence of relevant ocular and/or oculomotor disturbances.

For each identified saccade, the amplitude (*A*), duration (*D*), latency, and peak velocity (*V*_*p*_) were calculated. *A*/*D* and *A*/*V*_*p*_ relationships (main sequence) were evaluated and best fitted. For the *A*/*D* relation a linear regression (*D* = m*A + *q*) was used, while for the *A*/*V*_*p*_ relation the fitting curve was derived from the function *V*_*p*_ = 1/(*α* + *β*/*A*). The *K* (mean velocity/peak velocity ratio) and Skewness (saccadic rise time/duration ratio) parameters, able to provide a description of velocity responses, were also evaluated [25, 26], and their relationships with the Amplitude (*A*/*K*, *A*/Skewness) were examined by linear best fitting (*K* = *m* k*A + *q* *k*; Skewness = *m* s*A + *q* *s*) [26].

At a first examination of the acquired traces, a worsening was evident in the second test performed by F. I., with some SIF tracts and frequent blinks; a recovery was instead evident in the last recording.

The saccadic parameters, resulting from all the six registrations, are summarized in Table 1, where the normal values (obtained from a sample of 10 normal adult subjects performing the same test) are also indicated.

While A. I. presents normal values in the three tests, F. I. shows a significant alteration of the main sequence at the second test, characterized by saccadic slowing (lower 1/*α* values in the *A*/*V*_*p*_ relationship, *P* < .05) with duration increase (greater *m* slope in the *A*/*D* relationship, *P* < .01) and a subsequent improvement after miglustat therapy. Moreover, from 2000 to 2007 she presents a large reduction in *m* *k* slope and an increase in *m* *s* slope in the *A*/*K* and *A*/Skewness relationships, respectively, even though their values appear within the normal confidence interval (Table 1).

## 3. Discussion

The two patients were initially treated with an ERT dosage for GD1, because epilepsy was considered a casual event not correlated to the CNS glycolipid storage observed in GD3, and the barbiturate therapy was effective. In fact, even if it is known that barbiturate treatment could affect eye movements, in F. I. the therapy was always the same; therefore, it is unlikely that the eye movement changes could be due to barbiturate. In F. I., with more important visceral symptoms, ERT was started more precociously, but during the 2000–2005 period it was performed with considerable discontinuity because of the unaccepted administration route. At the second eye movement recording control (2005), some oculomotor signs of neurological involvement became manifest, especially the peak velocity reduction. The presence of oculomotor abnormalities is a sufficient condition to change the classification from nonneuronopathic to neuronophatic illness type, as indicated by Harris et al. [10] and Accardo et al. [26]. Her R353G/R353G genotype, already proposed as a neurological type [27] because of the presence of epilepsy, is confirmed as neurological owing to the onset of the new oculomotor alterations. We underline that in spite of the GD1 ERT dosage therapy, till about 40 years of age no evidence of neurological involvement (if we exclude epilepsy) was present; this indicates a low grade of neurological aggressiveness of this genotype. After two years of miglustat therapy, the oculomotor signs disappeared, showing the efficacy of SRT to improve (even by itself and not only if combined with ERT [23, 24]) some neurological symptoms. A positive result in one GD3 patient with myoclonic epilepsy was reported by Capablo et al. [23] using a combined ERT and SRT therapy. In our case SRT therapy alone did not influence the course of epilepsy, while it normalized saccades. On the contrary, a controlled trial in 30 GD3 patients [24], where miglustat was used in addition to ERT, did not show significant differences in the neurological signs (included saccadic eye movement characteristics) in a 24-month period, while a positive effect on systemic disease (pulmonary function and chitotriosidase activity) was observed. The vertical and horizontal saccadic velocities were significantly different between the GD patients and 10 age-matched control subjects at the starting point, but the standard deviation in the GD group was very large, therefore including cases with low velocities and other cases with normal ones. No significant variation was observed (in average) after miglustat therapy. Our case presented a clear peak velocity reduction, only 259 deg/s, which became 851 deg/s after two years of therapy (SRT alone), a very large variation (confirmed by two tests). To compare our results to other clinical cases, it would be interesting to know whether GD patients with the lowest saccadic velocities in the Schiffmann et al. series [24] clearly improved their ocular motor ability after miglustat therapy (together to ERT).

In A. I., the clinical picture appears unchanged in the 2005 and 2007 controls: the eye movements were normal as in 2000. In spite of the low dosage therapy and of the neurological genotype R353G/R353G [27], at an age of 38 years she did not yet show other neurological signs (in addition to the temporary epilepsy). Perhaps, the change of therapy (from ERT to SRT) did not permit any further mild neurological manifestation.

Concerning the presence of epilepsy, it was initially considered as a casual event in the two patients. After the appearance, in F. I., of new ocular motor pathological signs, it seemed more probable that epilepsy was part of the neurological picture of some GD3 patients. However, the inefficacy of the SRT therapy on EEG paroxysmal, with the necessity to maintain the barbiturate therapy, seems to indicate a low grade of brain activity of SRT, probably able to recover from only light and initial neurological manifestations.

Considering the clinical course in the two siblings, it can be hypothesised that the R353G homozygote genotype corresponds to a low-aggressiveness and late-onset GD3 illness.

In F. I., eye movement analysis permitted to point out the new slight neurological worsening, showing once more the value of this instrumental examination in monitoring the neurological situation, including the efficacy of therapy. In GD patients, beside eye movement examination, it is suitable to execute other exams able to investigate the brain-stem reticular formation, particularly those regarding the auditory pathways [28]. In fact, ocular motility is not the only neurological aspect showing up at the beginning of GD3, even if it is the most precocious [29]. Thus we recommend the convenience to exam periodically ocular motility in GD patients with risks genotypes to develop neurological symptoms.

It was previously reported that some GD1 patients present some slight eye movement alterations, related to saccadic velocity profiles [30], or evident neurological symptoms [17], bringing forward the concept of a phenotypic continuum from GD1 to GD3 classification. This concept is similar to the one that was already proposed by Goker et al. [31] for the heavy neurological involvements: a continuum between GD2 and GD3 classification. The results of the present study seem to be in agreement with this new approach to the evaluation of GD patients.

## Figures and Tables

**Table 1:** Eye movement characteristic parameters in the two GD sisters (A. I. and F. I.) compared to mean values and SD of 10 normal subjects (and corresponding confidence interval at *P* = .05). *A*/*D* *m*: slope of the amplitude/duration linear relationship; *A*/*D* *q*: intercept of the amplitude/duration linear relationship; *A*/*V*_*p*_ 1/*α*: velocity saturation value of the non linear amplitude/peak velocity relationship; *A*/*V*_*p*_ 1/*β*: slope at the lowest amplitude of the non linear amplitude/peak velocity relationship; *A*/*K* *m* *k*: slope of the linear amplitude/*K* ratio relationship; *A*/*S* *K* *m* *s*: slope of the linear amplitude/skewness relationship.

		*A*/*D* *m*	*A*/*D* *q*	*A*/*V*_*p*_ 1/*α*	*A*/*V*_*p*_ 1/*β*	Latency	*A*/*K* *m* *k*	*A*/*S* *K* *m* *s*
		ms/deg	*m* *s*	deg/s	1/s	*m* *s*	1/deg	1/deg
A. I.	2000	2.77	29	646	75	174	0.0041	−0.028
	2005	2.61	31	628	63	198	0.0040	−0.026
	2007	2.48	33	881	69	173	0.0015	−0.005

F. I.	2000	2.69	46	570	56	191	0.0031	−0.019
	2005	8.77	50	259	56	203	−0.0002	−0.0006
	2007	2.52	30	851	53	199	−0.0007	0.0014

Normal Subjects	mean	1.86	36	1282	61	188	0.0023	−0.013
	SD	0.47	5	355	15	36	0.0022	0.011

Conf. Int. (*P* = .05)	min	0.86	25	524	28	111	−0.003	−0.036
	max	2.86	47	2040	93	265	0.0072	0.01
